# ACVR mutation [NM_001105.4:c.774G>T (p.Arg258Ser)] in pediatric fibrodysplasia ossificans progressiva complicated by scoliosis: a case report

**DOI:** 10.3389/fped.2025.1608503

**Published:** 2025-12-15

**Authors:** Fangjun Yang, Miaomiao Liu, Shengtai Pei, Jin Huang, Huaming Wang

**Affiliations:** The Second Department of Pediatric Orthopedics, Gansu Provincial Hospital of Traditional Chinese Medicine, Lanzhou, China

**Keywords:** fibrodysplasia ossificans progressiva, heterotopic ossification, ACVR1 mutation, early diagnosis, scoliosis

## Abstract

This case report describes a 5-year-old female with fibrodysplasia ossificans progressiva (FOP), a rare and debilitating genetic disorder characterized by progressive heterotopic ossification. Initially misdiagnosed as an “aggressive chondroma” after two surgical resections, the patient developed recurrent paravertebral and iliopsoas ossification, culminating in severe functional impairment. Genetic testing confirmed a *de novo* pathogenic variant in the ACVR1 gene (NM_001105.4:c.774G>T; p.Arg258Ser). Management with NSAIDs and glucocorticoids yielded significant symptomatic improvement. This case underscores the critical importance of early genetic diagnosis to avert iatrogenic harm and guide appropriate conservative management.

## Introduction

Fibrodysplasia ossificans progressiva (FOP) is an extremely rare and disabling genetic disorder characterized by progressive heterotopic ossification (HO) of soft tissues, leading to ectopic bone formation in extraskeletal sites, alongside congenital malformations of the great toes (1, 2). Epidemiological studies indicate a prevalence of approximately 1 in 2,000,000 individuals, with an estimated 500–600 affected patients in China (3). The definitive molecular diagnosis of FOP relies on DNA sequence analysis of the ACVR1 gene (4). The pathogenesis of HO in FOP is primarily driven by dysregulated bone morphogenetic protein (BMP) signaling due to mutations in the activin receptor type I/activin-like kinase 2 (ACVR1/ALK2) (5). More than 95% of classic FOP cases harbor the recurrent c.617G>A (p.R206H) variant in ACVR1 (6). Here, we present the case of a female patient whose clinical symptoms began at age 5, with a definitive genetic diagnosis established later. Genetic analysis identified a heterozygous ACVR1 mutation (NM_001105.4:c.774G>T; p.Arg258Ser), resulting in an arginine-to-serine substitution at position 258 within the protein kinase domain. This report discusses the pathogenetic mechanisms, clinical spectrum, and diagnostic criteria of FOP, while emphasizing the importance of heightened clinical awareness and comprehensive patient and family education.

## Clinical case report

A 5-year-old female was initially evaluated in April 2020 for chronic low back pain persisting for four months. Radiologic studies revealed localized increased bone density. An excisional biopsy performed at a local hospital, under the preliminary suspicion of a neoplastic bone lesion, showed histopathological features of hypercellular cartilage and immature osteoid formation, interpreted as an “aggressive chondroma.” No further genetic or specialized immunohistochemical analysis was conducted.

In October 2020, a rapidly enlarging, firm dorsal mass emerged at the previous surgical site, prompting a second surgical resection. The recurrent lesion was again interpreted as consistent with chondromatous proliferation, without malignant features. The patient experienced only temporary symptomatic relief postoperatively.

**Figure 1:**
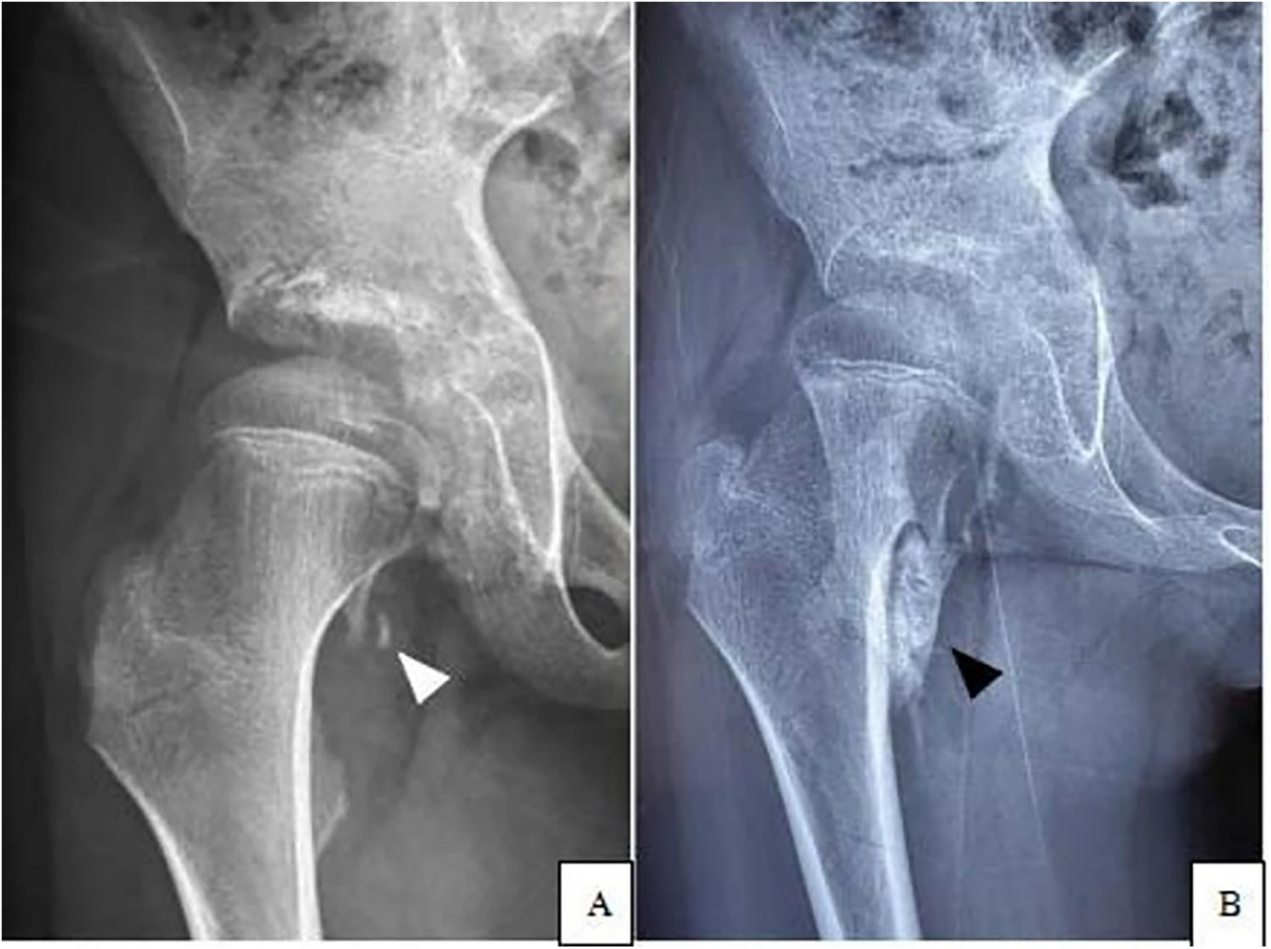
Hip radiographs demonstrating progressive heterotopic ossification inferomedial to the right femoral neck. **(A)** Examination performed in November 2023 shows early heterotopic ossification (white triangle). **(B)** Follow-up image from April 2024 reveals significant enlargement of the heterotopic bone (black triangle).

By November 2023, the patient developed progressive right hip pain and significantly restricted mobility, necessitating referral to our institution. Initial hip radiography revealed minor heterotopic ossification inferomedial to the right femoral head (Figure 1A). Given the subtlety of the findings and absence of acute inflammation, a conservative approach was adopted.

**Figure 2:**
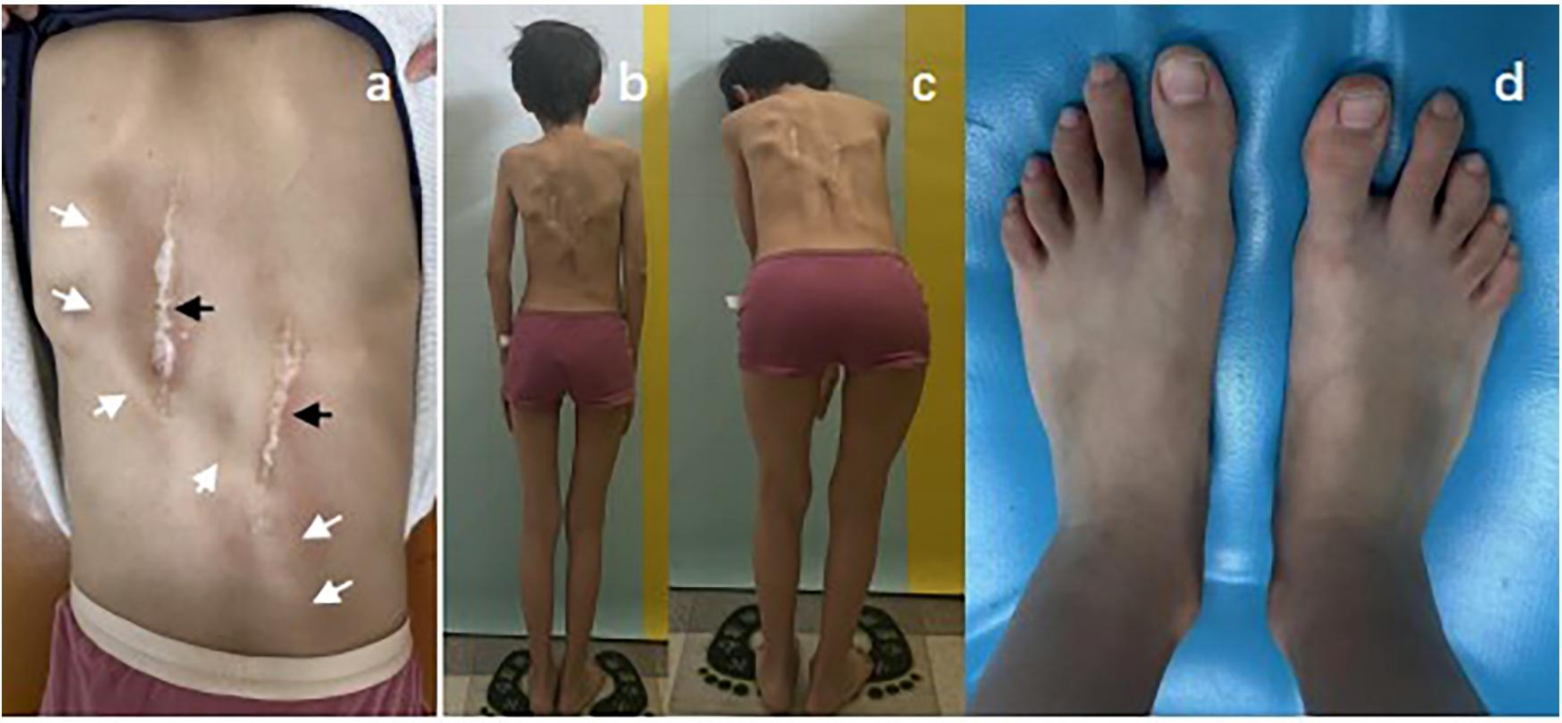
Clinical manifestations of the patient. **(a)** Soft tissue induration over the dorsal region indicative of ectopic ossification (white arrow) and surgical scar (black arrow). **(b)** Scoliosis evident on physical examination. **(c)** Marked limitation in trunk mobility. **(d)** Absence of great toe deformity.

In April 2024, the patient returned with markedly worsened right hip stiffness and persistent pain, significantly impairing ambulation and daily activities. Follow-up radiographs demonstrated progression of the heterotopic ossification around the right femoral head compared to the previous study (Figure 1B). Physical examination revealed two well-healed surgical scars over the lumbosacral region (Figures 2a–c). Palpation disclosed firm, fixed subcutaneous ossifications within the paraspinal soft tissues. Lumbar spine mobility was globally reduced. Notably, the bilateral great toes exhibited normal morphology without classic valgus deformity or symphalangism (Figure 2d). Neurological examination of the lower limbs was intact.

**Figure 3:**
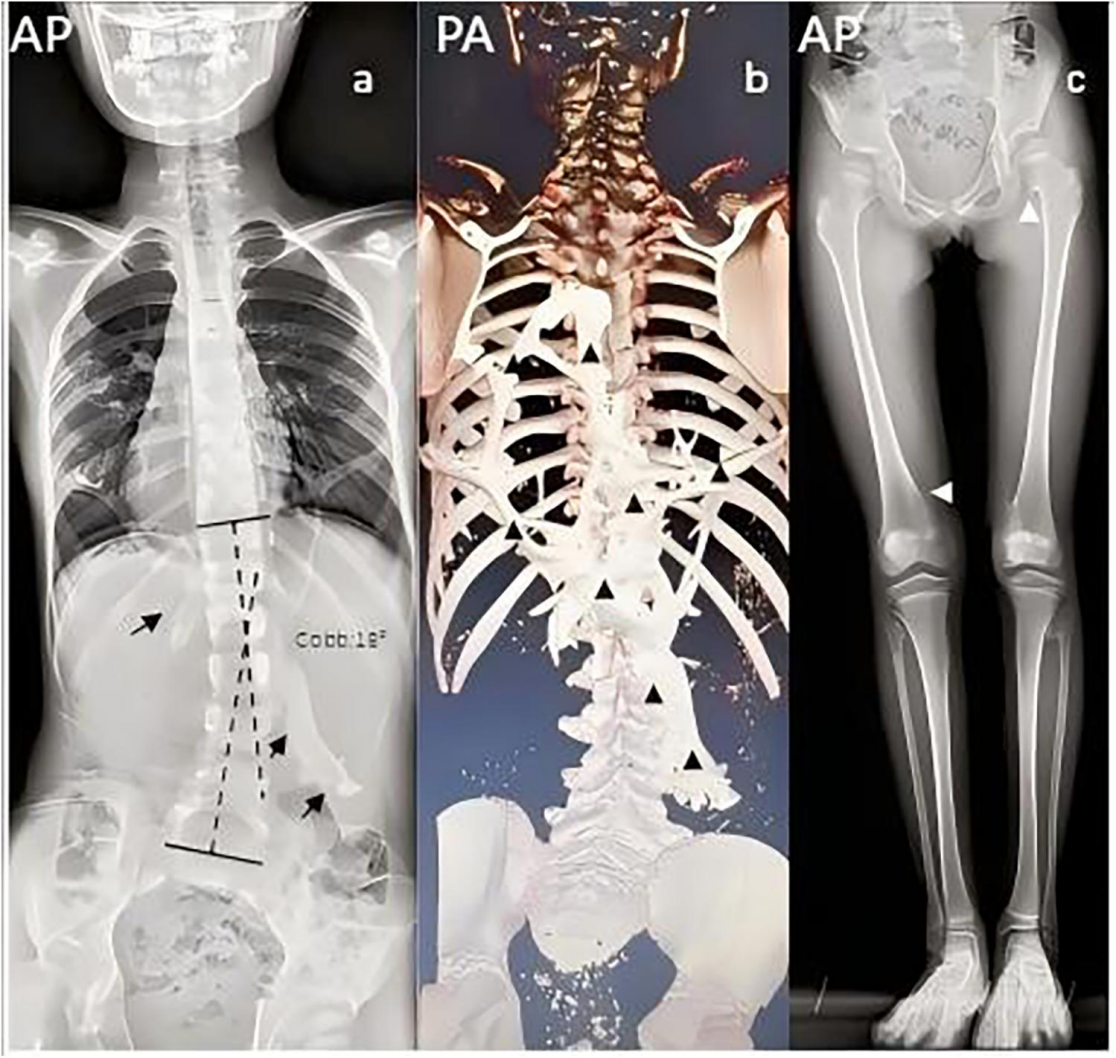
Imaging features of heterotopic ossification and skeletal abnormalities. **(a)** Anteroposterior spinal radiograph (April 2024) shows extensive paravertebral heterotopic ossification (black arrows) associated with scoliosis (Cobb angle, 18°). **(b)** Lateral 3D CT reconstruction of the thoracolumbar spine (April 2024) demonstrates sheet-like heterotopic bone formation within the dorsal fascia (black arrow). **(c)** Full-length anteroposterior radiograph of the lower limbs (April 2024) reveals multiple exostosis-like protrusions (white triangles) at the proximal femora, medial distal femora, and lateral proximal tibiae. The Risser sign is grade 0. Images are not to scale.

**Figure 4:**
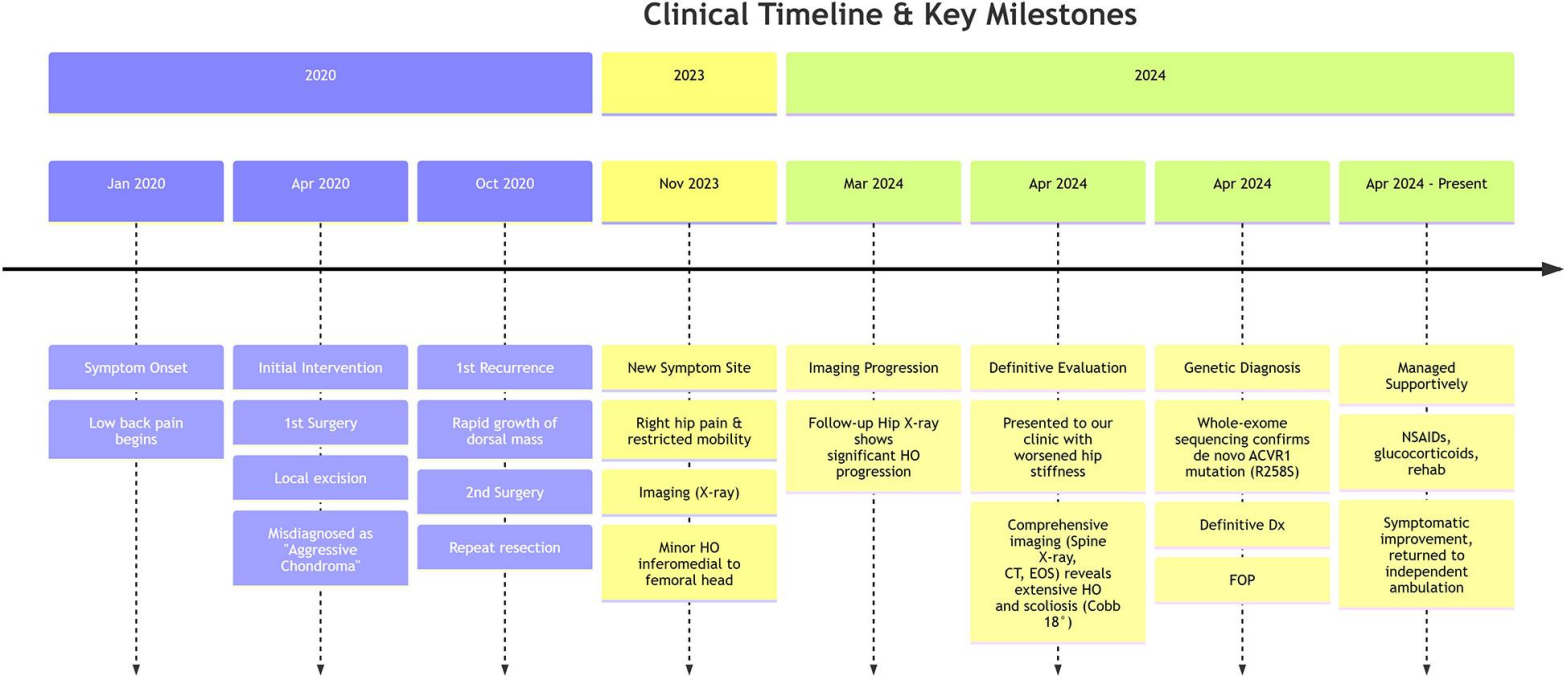
Clinical timeline from symptom onset to definitive diagnosis. HO, Heterotopic Ossification; FOP, Fibrodysplasia Ossificans Progressiva.

Anteroposterior spinal radiographs revealed extensive radiopaque lesions in the dorsal paraspinal regions, associated with scoliosis (Cobb angle 18°) (Figure 3a). CT with three-dimensional reconstruction confirmed scoliotic deformity caused by extensive heterotopic ossification within the dorsal fascia of the thoracolumbar region (Figure 3b). Standing full-length radiographs (AP) showed exostosis-like lesions bilaterally at the proximal femora, medial distal femora, and lateral proximal tibiae (Figure 3c). The Risser sign was grade 0. Figure 4 illustrates the clinical timeline. Readers are advised that the presented full-length radiographs are composite images and are not at a uniform scale; interpretation of absolute dimensions should therefore be made with caution. All clinical measurements were performed using the calibrated PACS system tools.

## The patient's perspective

The diagnostic odyssey caused significant familial distress. The child's chronic pain, functional limitations, and mass recurrence after two surgeries created immense anxiety and uncertainty. The parents expressed profound frustration over the inability to secure a definitive diagnosis, hoping primarily to understand the cause to prevent further harm. Upon receiving the confirmed genetic diagnosis of FOP, the family, while saddened by the progressive prognosis, felt a sense of clarity and closure. This definitive answer empowered them with a clear management strategy centered on avoiding iatrogenic trauma. Looking ahead, their concerns are focused on managing pain flare-ups, preserving mobility and independence, and maintaining quality of life, including school attendance and peer engagement. They voiced a strong hope for future targeted therapies.

## Genetic findings

After obtaining informed parental consent and ethics approval (Gansu Provincial Hospital of TCM, 2025-081-01), peripheral blood samples were collected from the patient and both parents for genetic testing. Genomic DNA was extracted, fragmented, and used to prepare sequencing libraries. Target enrichment was performed using the Roche KAPA HyperExome kit. High-throughput sequencing was conducted on the MGISEQ-2000 or DNBSEQ-T7 platforms. Bioinformatic analysis was performed to identify sequence variants. Quality control metrics showed a mean sequencing depth of 200× in target regions, with >98.5% of targeted bases covered at >20×.

Whole-exome sequencing (WES) of the proband-parent trio identified a heterozygous missense variant in the ACVR1 gene (NM_001105.4: c.774G>T; p.Arg258Ser) in the proband at genomic position chr2:158626896 (GRCh37). Sanger sequencing confirmed the variant was *de novo*, as both parents were wild-type. The variant results in an arginine-to-serine substitution at position 258 within the kinase domain (Table 1).

**Table 1:** Whole-exome sequencing and variant analysis summary.

Category	Parameter	Proband (II-1)	Father (I-1)	Mother (I-2)
Sample information	Sample ID	130U053175	130U053176	130U053177
	Sex	Female	Male	Female
	Sample Type	Whole Blood	Whole Blood	Whole Blood
Sequencing metrics	Raw Data Output (Mb)	32,496.69	26,024.87	18,229.28
	Target Region Coverage	100%	100%	99.99%
	Mean Depth of Coverage (X)	459.24	352.90	249.27
	>10× Coverage (%)	99.86%	99.98%	99.82%
	>20× Coverage (%)	99.78%	99.95%	99.76%
Variant findings	Gene	ACVR1	—	—
	Genomic Coordinate (GRCh37)	chr2:158626896	—	—
	Transcript/Protein Change	NM_001105.4: c.774G>T/p.Arg258Ser	Wild-type	Wild-type
	Zygosity	Heterozygous	Wild-type	Wild-type
	Variant Ratio (Ref/Alt)	216/144	—/—	—/—
	Inheritance	*De Novo*	—	—
Variant interpretation	Disease (OMIM)	Fibrodysplasia Ossificans Progressiva (#135100)	—	—
	Inheritance Pattern	Autosomal Dominant (AD)	—	—
	ACMG Classification	Likely Pathogenic	—	—
	Applied ACMG Codes	PS2, PS4_supporting, PM2, PP3, PP4	—	—

Per ACMG guidelines, this variant was classified as Likely Pathogenic based on the following criteria: (PS2) *de novo* occurrence; (PS4_Supporting) Previous reports in unrelated FOP patients; (PM2) Absent in population databases; (PP3) Multiple computational predictions support a deleterious effect; (PP4) Highly specific clinical presentation for FOP (Table 2).

**Table 2:** Supporting evidence for the pathogenicity assessment of the ACVR1 variant.

Evidence type	Result	Interpretation
Population frequency	GnomAD, ExAC, ESP6500, 1000 Genomes: Not found (or extremely low frequency)	Supports pathogenicity (PM2)
Computational evidence	In Silico Prediction: MutationTaster: Damaging Revel: Damaging SIFT: Damaging GERP++: Conserved PhyloP: Conserved	Strong computational support for deleterious effect (PP3)
Functional evidence	Literature: Reported in multiple unrelated FOP patients (PMIDs: 22,796,417, 27,081,558, 38,664,849)	Supports pathogenicity (PS4_supporting)
Segregation evidence	*De Novo*: Confirmed via parental testing	Very strong evidence for pathogenicity (PS2)
Phenotypic evidence	Patient's phenotype (limitation of joint mobility, bony masses) is highly specific to FOP	Supports pathogenicity (PP4)

This variant is conclusively associated with FOP (OMIM #135100). The identification provides a definitive molecular diagnosis. No other reportable variants were detected.

## Management and follow-up

During acute flare-ups, symptomatic management involved oral NSAIDs and corticosteroids to alleviate inflammation, alongside physical therapy. In the chronic phase, invasive procedures (surgical excision, biopsies, intramuscular injections) were strictly avoided to prevent exacerbation (Table 3). At the time of manuscript submission, the patient showed marked clinical improvement, with significant resolution of right hip pain, regained ambulatory function, and a return to regular school activities.

**Table 3:** Summary of therapeutic interventions for the patient.

Therapy category	Specific regimen	Duration/frequency	Efficacy & tolerability
NSAIDs	Ibuprofen 10 mg/kg/dose	Every 6–8 h as needed	Well tolerated with adequate symptomatic relief
Corticosteroids	Prednisolone 1 mg/kg/day (max 40 mg/day)	5–7 days with 1–2 week taper during flares	Effective in reducing acute inflammation
Physical Therapy	Outpatient ROM and strength training	2–3 times/week for 6-month periods	Excellent adherence

ROM, range of motion.

All interventions were well tolerated without reported adverse effects.

## Discussion

FOP is a rare autosomal dominant disorder characterized by progressive HO and congenital skeletal anomalies, most classically great toes malformations. The mean age of symptom onset is approximately 5.4 years, with initial HO typically developing axially before progressing caudally and distally (7–9). Atypical features can include spinal deformities, osteochondromas, and sensorineural hearing loss (10, 11).

Notably, our patient lacked classic hallux valgus (Figure 2d), highlighting phenotypic variability and the risk of diagnostic delay. Her initial presentation involved lower back pain from undiagnosed HO, tragically leading to two surgical interventions.This diagnostic odyssey and the patient's unique clinical features prompted a thorough genetic investigation to elucidate the underlying cause.

The molecular understanding of FOP has advanced significantly, establishing that the disorder is driven by gain-of-function mutations in the ACVR1 gene, leading to constitutive BMP pathway signaling and aberrant osteogenesis (12–14). Dysregulated SMAD signaling further contributes to this pathologic process (15). Against this mechanistic backdrop, the genetic analysis of our patient revealed the p.Arg258Ser variant, enabling a deeper exploration of its distinct characteristics.

Our patient's presentation underscores the phenotypic heterogeneity associated with ACVR1 mutations beyond the classic p.Arg206His. The p.Arg258Ser variant identified in our case, while also located in the GS domain and conferring a gain-of-function, demonstrates distinct characteristics. Structurally, the alteration at residue Arg258 may result in subtler changes to receptor regulation compared to Arg206, potentially explaining the lower penetrance of great toe malformations observed in our patient and other reported p.Arg258Ser cases. Phenotypically, this variant appears to be associated with a prominent axial skeleton phenotype, as evidenced by the severe, early-onset scoliosis in our case—a feature less emphasized in the classic R206H profile. This suggests a potential genotype-phenotype correlation where the p.Arg258Ser variant defines a clinical subtype with a higher propensity for significant spinal involvement.

Given this phenotypic variability, a lack of disease awareness frequently leads to misdiagnosis (e.g., as sarcoma or fibromatosis) (16), which can result in harmful interventions, disease exacerbation, and irreversible mobility loss (17). An international survey found 87% of patients were initially misdiagnosed, with 67% undergoing harmful procedures (18). A Chinese study reported an 84.7% misdiagnosis rate and a 6.8 year average diagnostic delay (19). These findings underscore the need for improved clinician education. While clinical diagnosis can be sufficient for classic presentations, genetic confirmation is crucial in atypical or early-stage cases like ours.

Trauma (surgery, biopsies, injections) is a well-established trigger for HO flare-ups (13, 20). Therefore, establishing an early and accurate diagnosis is essential to prevent iatrogenic harm. Our patient presented around age five with dorsal nodules, consistent with typical onset and axial predilection (21, 22), later progressing to the hips. Multi-site imaging demonstrating HO (Figure 3) raised diagnostic suspicion, confirmed genetically.

Currently, no cure exists for FOP. Management is supportive, focusing on symptom relief and maintaining mobility via adapted physical therapy (23). Emerging strategies, including gene-based and biologic therapies, are under investigation (24–26). Treatment personalization remains challenging. In a relevant case, Sun et al. (27) reported successful resection of iliopsoas HO in a 13-year-old with FOP followed by indomethacin, suggesting that in highly specific and carefully selected cases, surgical intervention might be considered. Nevertheless, multidisciplinary management and trauma avoidance remain the cornerstones of management to preserve quality of life.

## Conclusions

In conclusion, FOP requires early recognition of its clinical features, even before overt heterotopic ossification appears. Diagnosis should be confirmed genetically to avoid misdiagnosis and unnecessary harmful procedures, especially surgery. Treatment is supportive, emphasizing patient education to prevent trauma-induced flare-ups.

## Data Availability

The original contributions presented in the study are included in the article/Supplementary Material, further inquiries can be directed to the corresponding authors.
